# Annual Meetings of Hospitals

**Published:** 1921-04-09

**Authors:** 


### Annual Meetings of Hospitals.

### Western Ophthalmic Hospital.

The sixty-fifth annual report of the Western Ophthalmic Hospital, presented at the annual meeting held recently, states that the total income for 1920 was £4,730, as compared with £3,706 in 1919, and exceeded the expenditure by £605. In-patients' payments totalled £378, and exceeded those of 1919 by £61. Out-patients' payments amounted to £327, an increase of £47 for the same period. By the end of last year 949 school children had attended the clinic, which, at the special request of the L.C.C., was opened in April last. Plans have been drawn up for the rebuilding of the hospital, towards the cost of which the King's Fund have offered £2,000 on condition that a further £2,000 is raised by March 31, 1921.

### Hospital for Diseases of the Throat.

The fifty-eighth annual meeting was held on March 23, Mr. James Jackson, J.P., in the chair. The report states that the amalgamation with the London Throat Hospital has been completed, and the combined hospitals have been working at Golden Square as from September 1, 1920. With the exception of the closing of the institution for necessary cleaning and repairs during August, the work has been continuous throughout the year. The number of in-patients treated was 1,631, compared with 1,441 in 1919. The new out-patients in 1920 totalled 14,491, against 13,205 during the previous year. Owing to the long distances from which many of the patients come, and the increased cost of travelling, the medical staff had, where possible, prescribed medicine, etc., for more than the usual weekly attendance, the total of such extra weeks being 25,410, but in calculating "the weekly cost of each out-patient attendance" for the various hospital funds, it is not permissible to take this excess medicine into account. That to a great extent explains the heavy increase in the accounts under the head of "Surgery and Dispensary," which last year cost £1,862. The total income for the year was £13,043, and exceeded the expenditure by £2,625. Efforts to dispose of the Golden Square site, with a view to rebuilding the hospital in the suburbs, have proved abortive, and it has been decided to improve existing premises by rebuilding Nos. 22 and 24 Beak Street, thus providing better accommodation for the nurses and domestic staff, and to continue the hospital work on the present site. Plans and estimates of the proposed improvements will shortly be submitted to the King's Fund for approval.

